# Molecular mapping of the Cf*-10* gene by combining SNP/InDel-index and linkage analysis in tomato (*Solanum lycopersicum*)

**DOI:** 10.1186/s12870-018-1616-7

**Published:** 2019-01-08

**Authors:** Guan Liu, Tingting Zhao, Xiaoqing You, Jingbin Jiang, Jingfu Li, Xiangyang Xu

**Affiliations:** College of Horticulture and Landscape Architecture, Northeast Agricultural University, Mucai Street 59, Xiangfang District, Harbin, 150030 China

**Keywords:** Tomato, *Cladosporium fulvum*, Cf*-10* gene, QTL mapping, SNP-index, InDel-index, KASP

## Abstract

**Background:**

Leaf mold, one of the major diseases of tomato caused by *Cladosporium fulvum* (*C. fulvum*), can dramatically reduce the yield and cause multimillion dollar losses annually worldwide. Mapping the resistance genes (*R* genes) of *C. fulvum* and devising MAS based strategies for breeding new cultivars is an effective approach to improve the resistance in tomato. Up to now, many *C. fulvum* genes or QTLs have been mapped using different genetic materials, but few studies focused on Cf*-10* gene positioning.

**Results:**

In this study, we investigated the genetic rules for Cf*-10* and used a novel combinatorial strategy to rapidly map the Cf*-10* gene. Initially, the performance of F_1_, F_2_ and BC_1_F_1_ individuals after infection, demonstrated that the resistance against *C. fulvum* was controlled by a single dominant gene. Two pools of resistant and susceptible individuals from F_2_ population were investigated, using mapping by sequencing approach and Cf*-10* was found to be localized to 3.35 Mb and 3.74 Mb on chromosome 1, employing SNP/InDel index methods, respectively. After accounting for overlapping regions, these two algorithms yielded a total length of 3.29 Mb, narrowing down the target region. We further developed five serviceable KASP markers for this region based on sequencing data and conducted local QTL mapping using individuals from the F_2_ population, except for mapping by sequencing as mentioned above. Finally Cf*-10* gene was mapped spanning a region of 790 kb, where only one gene (*Solyc01g007130.3*) was annotated as probable receptor protein kinase TMK1 with a LRR motif, a common R gene characteristic. The RT-qPCR analysis further confirmed the localization and the relative expression of *Solyc01g007130.3* in Ontario 792 and was found to be significantly higher than that in Moneymaker at 9 dpi and 12 dpi, respectively.

**Conclusion:**

This study proposed a novel combinatorial strategy by combining SNP-index, InDel-index analyses and local QTL mapping using KASP genotyping approach to rapidly map genes responsible for specific traits and provided a robust base for cloning the Cf*-10* gene. Furthermore, these analyses suggest that *Solyc01g007130.3* is a potential candidate to be regarded as Cf*-10* gene.

**Electronic supplementary material:**

The online version of this article (10.1186/s12870-018-1616-7) contains supplementary material, which is available to authorized users.

## Background

Cloning genes associated with important agronomic traits is one of the major components of plant functional genomics research. Map-based cloning is an important and reliable method, and many genes with vital functions from economically important crops [1–5] and fruit trees [6, 7] have been cloned, primarily using simple sequence repeat (SSR), sequence-characterized amplified region (SCAR) markers and/or cleaved amplified polymorphic sequence (CAPS) based approaches. A number of genes or quantitative trait loci (QTLs) such as *ER4.1* [8], *dl* [9], *Ty-2* [10], *ms10* [11], *fw2.2* [12] and *fw11.3* [13] were fine mapped and isolated from tomato using SSR/InDel makers. However, the markers and CAPS derived from single nucleotide polymorphisms (SNPs) to clone these genes often results in low resolution and are time-consuming. Mapping and cloning of genes quickly is a major challenge in the post-genomic era. However, next-generation sequencing (NGS) technologies provide an effective solution to the problem and a number of genes were fine mapped and cloned by employing high-density genetic map based approaches. A high-density genetic map constructed by using Illumina-based whole genome re-sequencing of soybean genome, a 29.7 kb QTL containing two candidate genes involved in resistance against southern root-knot nematode (RKN) was mapped on chromosome 10 [14]. Another research highlighted the mapping of QTL-*U5* responsible for fruit colour in cucumber spanning a region of 313.2 kb located on chromosome 5 containing 320 genes, of which 39 were synonymous [15]. Mapping by sequencing or bulked segregant analysis (BSA) is another method for rapid gene mapping [16, 17]. Pandey et al. [18] identified a region of 3.06 Mb on pseudomolecule A03 responsible for rust and late leaf spot (LLS) resistance using whole-genome re-sequencing QTL-seq approach. The study further identified that about 25 candidate genes were affected by 30 non-synonymous SNPs besides other nine candidate genes affected by three synonymous SNPs responsible for rust and LLS resistance, respectively. The RNA Seq-BSA was also adopted to identify SNPs associated with *Yr15*, involved in resistance against yellow rust in wheat [19]. Several other studies used similar methods to fine map genes for different economically important traits [20–22].

Plants have evolved a plethora of resistance strategies in response to the invasion of pathogens [23–26], and most prominent among them encompasses R gene induced recognition, exploiting nucleotide-binding sites and leucine-rich repeat (NBS-LRR) domains to trigger the disease resistance response [27, 28].

*C. fulvum* series genes are special *R* genes that play important roles in resisting tomato leaf disease caused by *Cladosporium fulvum*, which can cause serious damage to tomato yield and quality. To date, many *C. fulvum* genes have been mapped in different genomic regions, but most of these *C. fulvum* genes are located at the Milky Way site of chromosome 1 such as, Cf*-4,* Cf*-9, Hcr9-4E, Hcr9-9B,* Cf*-ECP1,* Cf*-ECP2,* Cf*-ECP3,* Cf*-ECP4,* Cf*-ECP5* and Cf*-19* genes [29–38]. Whereas, other *C. fulvum* genes, such as Cf*-2,* Cf*-5* and Cf*-6*, were shown to be closely associated with chromosome 6 [39, 40]. Among all these genes, only Cf*-2,* Cf*-4,* Cf*-5*, Cf*-9* and Cf*-19* have been cloned and well characterized*.* All of these cloned Cf genes belong to one of the two multigene families designated as homologues of *Cladosporium fulvum* resistance genes (*Hcr*). The Cf*-9* and Cf*-2* genes consisting of 28 and 39 LRRs respectively, codes for transmembrane proteins [41, 42] while Cf*-5* gene has been predicted to encode a largely extracytoplasmic protein containing 32 LRRs. On the other hand, Cf*-4* encodes a membrane-anchored extracellular glycol-protein possessing only two LRRs which is much less than the LLRs encoded by Cf*-9* gene [31]. The candidate gene Cf*-19* (*Solyc01g006550.2.1*) encodes 30 LRRs, which is different from other *Hcr* genes. The C terminal of the protein encoded by Cf-4 gene was similar to those of Cf-9 and Cf*-19* genes [38, 39]. These genes seem to be evolutionarily conserved although, they have shown a certain degree of structural differences [38].

Previously, our research team had developed four molecular markers associated with Cf*-10* gene [43], but this is far from gene positioning or cloning. Recently, we investigated the expression patterns of differentially expressed genes (DEGs) in Ontario 792 harbouring Cf*-10* gene after infection [44]. In the current study, we explored the genetic characteristics of the Cf*-10* gene by developing a large F_2_ population and a BC_1_F_1_ population. By combining the SNP/InDel-index sequencing methods, we preliminarily mapped the Cf*-10* gene and then narrowed this key region by developing kompetitive allele-specific PCR (KASP) markers for the primary mapping region to genotype the remaining individuals of F_2_ population. The RT-qPCR was used to validate the expression of candidate genes. Our results provide a novel, rapid and labour-saving approach for mapping genes in general and form the basis of cloning and deciphering molecular dynamics of Cf*-10* gene in particular.

## Results

### The variation in disease resistance and test crosses

**Fig. 1:**
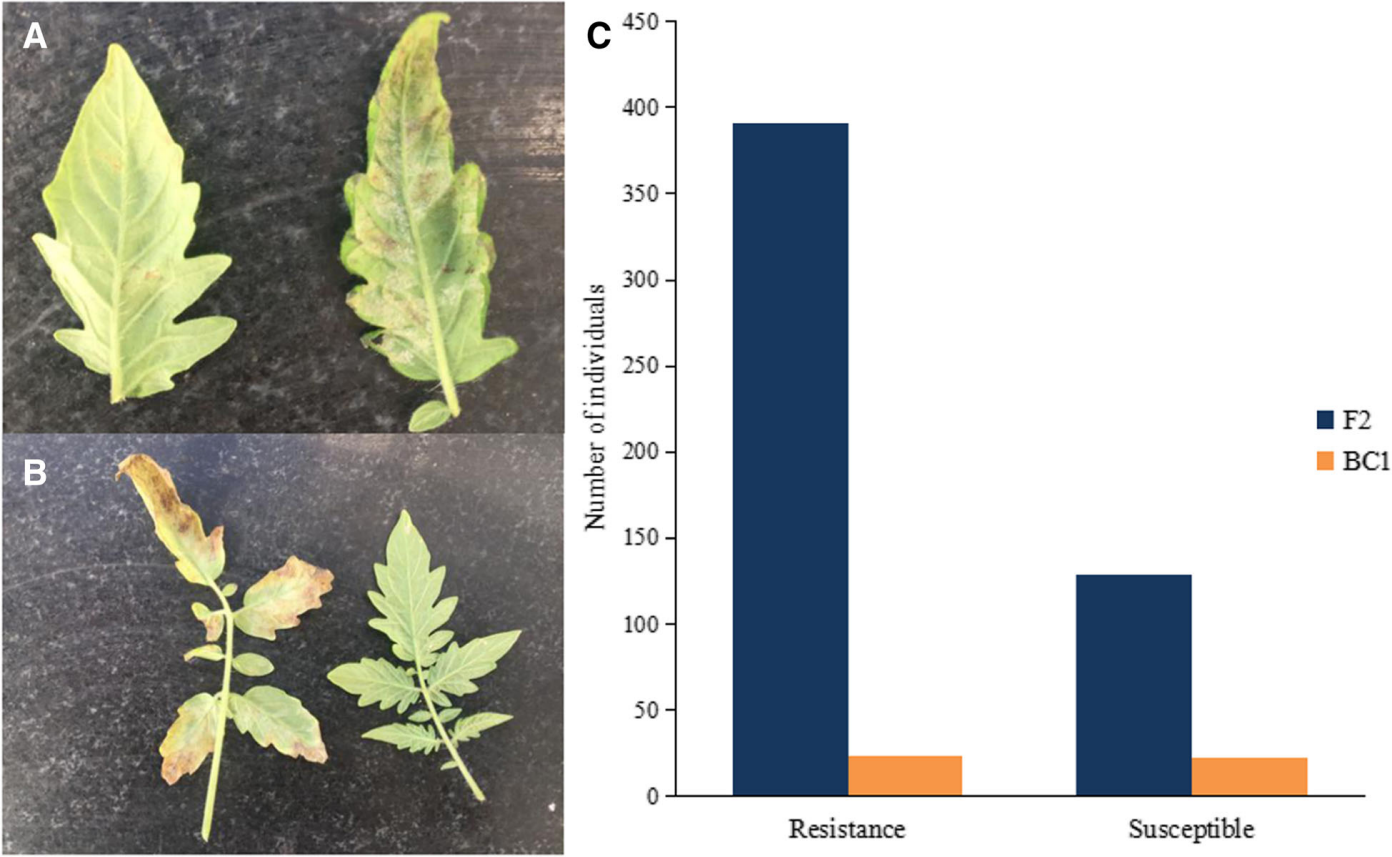
The phenotype and segregation patterns of tomato. **a** The performance of Ontario 792 (left) and Moneymaker (right) after inoculation at 21 dpi. **b** The performance of F_2_ individuals after inoculation at 21 dpi. The left leaf is susceptible, while the right one is resistant. **c** The distribution of resistant and susceptible plants in the F_2_ and BC_1_F_1_ populations

To investigate the genetic basis of Cf*-10*, we evaluated the disease resistance potential of Ontario 792, Moneymaker, F_1_, F_2_ and BC_1_F_1_ populations. Initially, we tested the resistance response of the two parents to ten different races (Table 1). Moneymaker demonstrated significantly higher susceptibility to all these races, while Ontario 792 was immune to race 1.2.4 and 1.4 besides being resistant to other eight races, indicating that Ontario 792 had broad-spectrum resistance against *C. fulvum*. Then we adopted the predominant physiological race 1.2.3.4 in Heilongjiang Province to test the performance of F_1_ and F_2_ individuals. To simplify the phenotype, plants with a disease score between 0 and 3 points were marked as resistant, while those with scores ≥5 points were marked as susceptible. All F_1_ plants showed resistance against the disease (Table 2), 391 individuals out of 529 from F_2_ population showed disease resistance while remaining 138 were found to be susceptible with a 3:1 segregation ratio as revealed by *Chi-square* test results (Fig. 1c), suggesting that Cf*-10* was controlled by a single dominant gene. This hypothesis was further supported by the BC_1_F_1_ population presenting a 1:1 Mendelian ratio (Fig. 1c; Table 2; χ^2^ = 0.011).

**Table 1:** The disease response of Moneymaker and Ontario 792 to different physiological races

Physiological races	Moneymaker		Ontario 792	
	Disease index	Resistance level	Disease index	Resistance level
1.2	65.4	HS	15.7	R
1.2.3	50.1	MS	20.1	R
1.2.3.4	55.5	HS	11.4	R
1.2.4	76.1	HS	0.0	I
1.3.4	58.4	HS	12.5	R
2.3	70.7	HS	22.6	R
1.3	59.9	HS	26.8	R
1.4	55.2	HS	0.0	I
1.2.3.4.5	56.1	HS	20.4	R
1.2.3.4.9	62.3	HS	15.3	R

*I* immune, *HR* highly resistant, *R* resistant, *MR* moderately resistant, *MS* moderate susceptibility, *HS* highly susceptible

**Table 2:** Genetic analysis of the Cf*-10* resistance gene in different populations

Generation	Total No. of plants	No. of resistant plants	No. of susceptible plants	The segregation ratio of R:S	χ^2^
Ontario 792	50	50	0	**–**	**–**
Moneymaker	50	0	50	**–**	**–**
F_1_	20	20	0	**–**	**–**
F_2_	529	391	138	2.83:1	0.082
BC_1_F_1_	45	23	22	1.045:1	0.011

χ^2^_0.05, 1_ = 3.84

### QTL-seq analysis combining SNP-index and InDel-index

A total of 116.30 Gb clean data were obtained by Illumina sequencing, including 62.61 Gb from the parents and 53.69 Gb from the two mixed pools, all of high quality (94.33% > Q30 > 91.25%) and with a stable GC content (36.77% > GC > 35.69%) (Table 3). The average sequencing depths for the two parents and the two F_2_ pools were 33.50 × and 29 ×, respectively. These high-quality data lay a robust foundation for subsequent analysis.

**Table 3:** An overview of the sequencing results

Sample	Clean Reads	Clean Bases (Gb)	Q30 (%)	GC (%)	Properly mapped (%)
R01	89,945,037	26.94	93.89	36.77	89.45
R02	119,076,889	35.67	91.25	35.69	89.53
R03	96,599,912	28.93	94.27	36.18	89.80
R04	82,672,515	24.76	94.33	36.30	90.47

**Fig. 2:**
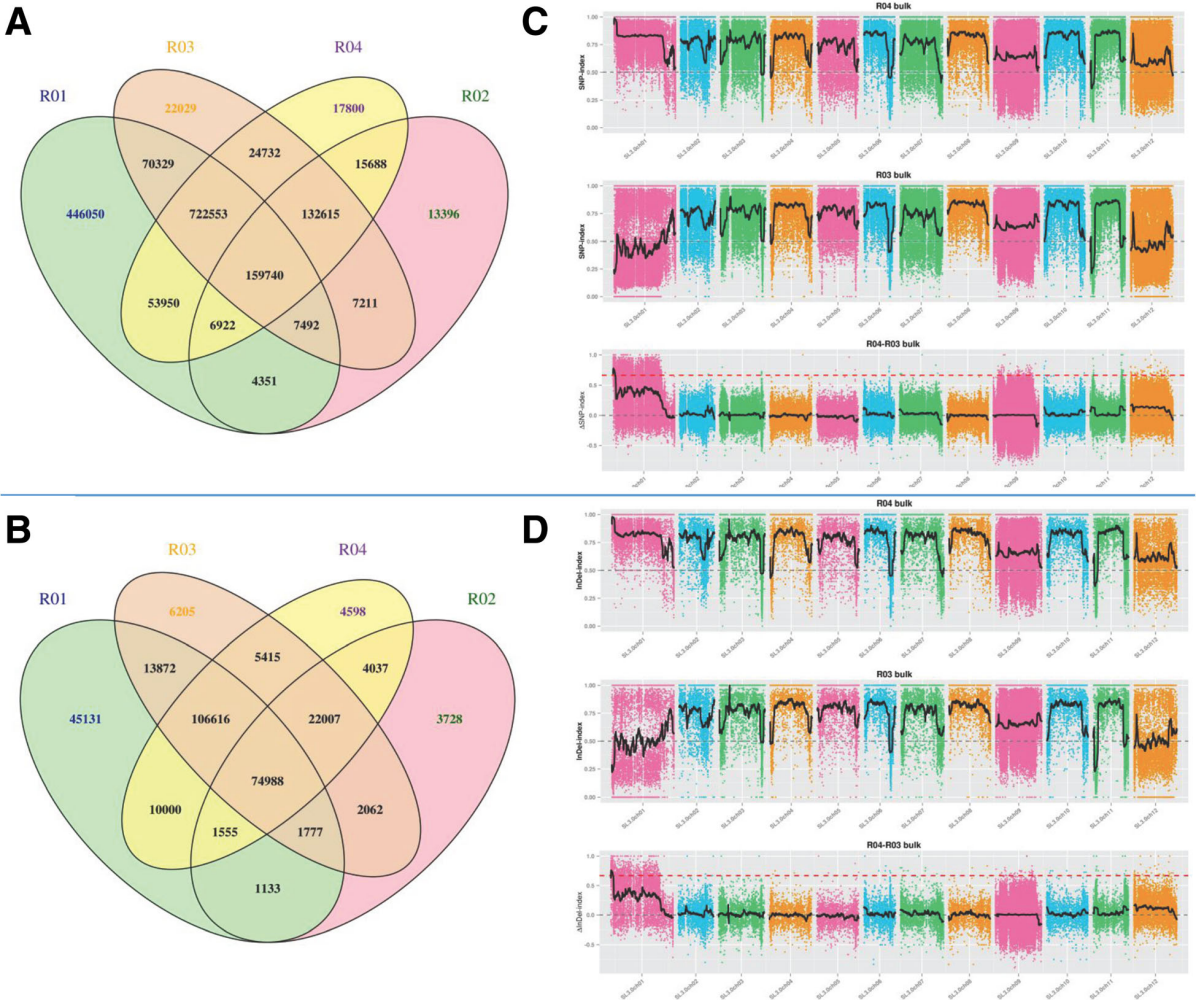
SNP statistics and BSA analysis. **a** Venn diagram of SNP in the four pools. **b** Venn diagram of InDel in the four pools. **c** SNP-index algorithm to map Cf*-10* gene. **d** InDel-index algorithm to map Cf*-10* gene

These reads were mapped onto the reference genome of *Solanum lycopersicum*. The average mapping rate was 96.61% for these four samples. A total of 1,465,985 SNPs were obtained from the parents, of which 10,293 SNPs were non-synonymous. A total of 202,784 SNPs were obtained from the two mixed pools. Notably, we detected 214,921 small InDels amongst the parents and 51,348 small InDels from the mixed pools. Venn diagrams were used to demonstrate the relationships between SNP and InDels among the parents and the mixed pools. As shown in the Venn diagrams, these four samples share 159,740 SNPs and 74,988 InDels respectively (Fig. 2a, b).

We used two different methods to map the QTLs responsible for Cf*-10* resistance. As shown in Fig. 2, only one QTL was identified and both the SNP-index and InDel-index association algorithms mapped this QTL to chromosome 1. More specifically, this QTL was located in the region 0–3,350,000 bp (3.35 Mb) using SNP-index method while InDel-index method revealed its presence in the region spanning 60,000–3,800,000 bp (3.74 Mb). By taking overlapping regions into account, these two methods yielded a total length of 3.29 Mb on chromosome 1. A total of 408 genes were annotated in this associated region, including 73 non-synonymous genes, 16 frameshifted genes and 16 genes with an extracellular LRR (eLRR) domain.

### Further mapping of the Cf*-10* gene

**Fig. 3:**
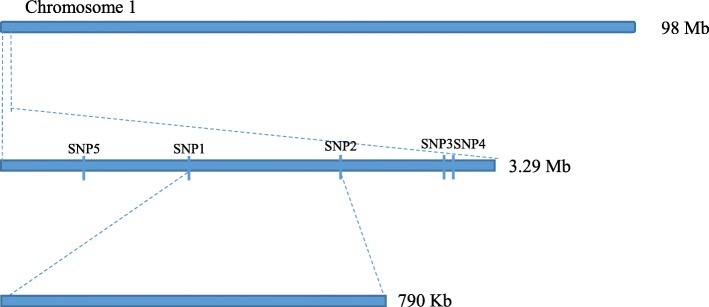
Molecular mapping of the Cf*-10* gene using SNP/InDel combinatorial approach

Since the 3.29-Mb region still contained a large number of genes, we developed 16 KASP markers, and five valid KASP markers in this region were used for genotyping an additional set of 147 individuals. The KASP genotyping results obtained from Ontario 792, Moneymaker and F_1_ individuals were consistent with our sequencing results, indicating that the sequencing and SNP calling results were reliable (Additional file 1: Table S5). Furthermore, we developed genetic map and also performed QTL mapping of this region so that the position of Cf*-10* resistance QTL was narrowed down and was located between SNP 1 and SNP 2, a 790-kb region (Fig. 3; Additional file 2: Figure S1). Fortunately, only the *Solyc01g007130.3* gene was annotated as probable receptor protein kinase TMK1 with a LRR motif, a common R gene characteristic suggesting that *Solyc01g007130.3* is a potential candidate to be regarded as Cf*-10* gene. Nevertheless, no SNPs or InDels were called in *Solyc01g007130.3* from Ontario 792 and Moneymaker according to our re-sequencing data. We further used Sanger sequencing to compare *Solyc01g007130.3* variation between Ontario 792 and Moneymaker and that no apparent differences were observed between them (data not shown).

### Screening of expression pattern of the candidate gene

**Fig. 4:**
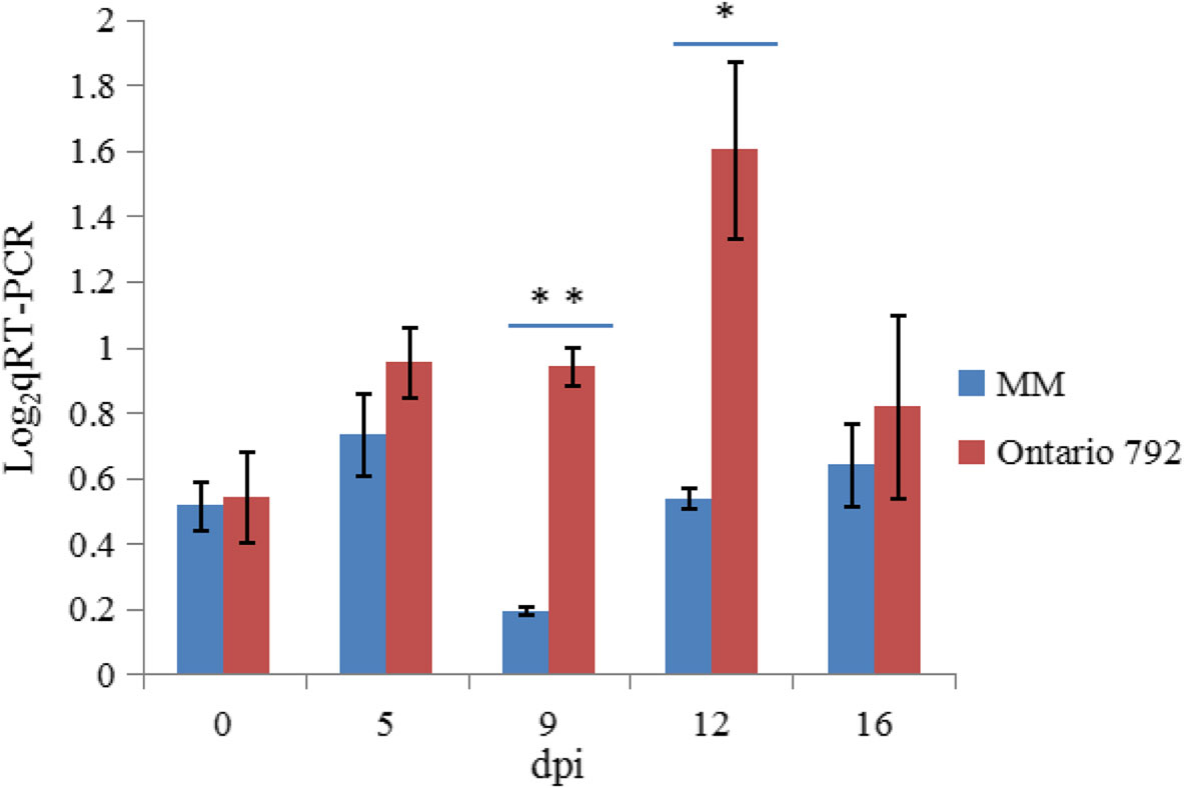
RT-qPCR analysis of *Solyc01g007130.3* in the QTL region on chromosome 1 in response to *C. fulvum* inoculation. Both Ontario 792 and Moneymaker were inoculated with *C. fulvum* for 5, 9, 12, and 16 d. The blue and red bars represent Moneymaker and Ontario 792, respectively. The results were statistically analysed using Student’s *t*-test (*, *P* < 0.05; **, *P* < 0.01) and the asterisks indicate that the difference in gene expression in Ontario 792 and Moneymaker plants was highly significant

We conducted a RT-qPCR assay to determine the expression response of *Solyc01g007130.3* in Ontario 792 and Moneymaker after inoculation. The RT-qPCR results demonstrated that the expression of *Solyc01g007130.3* was significantly induced in Ontario 792, except at 16 dpi (Fig. 4). No significant differences were observed between Ontario 792 and Moneymaker at 0 dpi, but at 9 dpi and 12 dpi, the relative expression of *Solyc01g007130.3* in Ontario 792 was significantly higher than that in Moneymaker. At 5 dpi and 16 dpi, the difference in expression was higher in Ontario 792 than in Moneymaker but was of lesser statistical significance. Considering these results together, we concluded that *Solyc01g007130.3* is a possible candidate for the gene underlying the QTL for Cf*-10* resistance.

## Discussion

### Rapid gene cloning from a large F_2_ population

Traditional map-based cloning is an efficient approach to isolate genes/QTLs responsible for desired agronomic traits [45–47]. Usually, a genetic map of an F_2_, double-haploid (DH) or recombinant inbred line (RIL) population based on hundreds of SSR or InDel markers is used to make a primary map, and then a near-isogenic line (NIL) is developed based on marker-assisted selection (MAS; newly discovered in the primary mapping region) to narrow down the region of interest to a sufficient size to screen for a few candidate genes. Unfortunately, this workflow always takes a relatively long time [48, 49]. Compared with genetic maps, the mapping by sequencing using NGS is a faster and reliable method for mapping QTLs [50]. Nevertheless, one mixed pool typically contains approximately 20–100 individuals and generally maps the target region at a Mb-level interval [19, 51–54] because of insufficient meiotic recombination events. We still have to perform fine mapping or use *omics* methods such as RNA-seq to further screen the candidate genes [55, 56].

KASP is one of the uniplex SNP genotyping platforms [57] and has been successfully used to genotype and map QTLs in bi-parental populations [58, 59]. Recently, Xu et al. [60] developed KASP markers spanning a 303 kb region mapped by a 50 + 50 mapping sequence strategy and genotyped 2274 F_2_ individuals. Finally, the region was narrowed down to 36.1 kb, and they identified a candidate gene, *ARN6.1*, responsible for waterlogging tolerance.

**Fig. 5:**
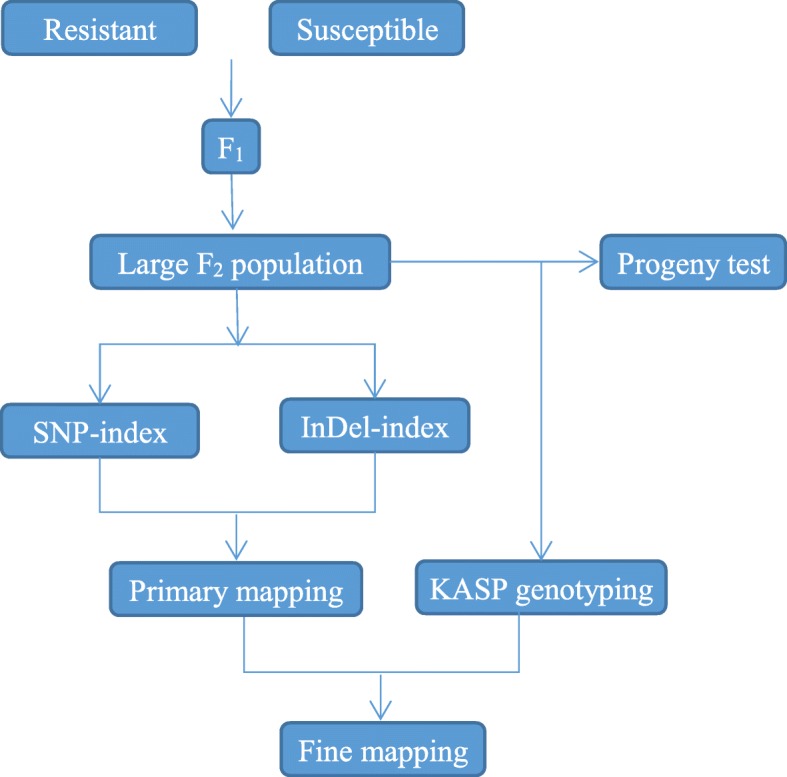
A novel combinatorial strategy to rapidly fine map genes

In the present study, we used mapping by sequencing by combining SNP-index and InDel-index analyses to primarily position the Cf*-10* gene to a 3.29 Mb region. Five KASP markers developed in this region helped us to narrow the Cf*-10* gene to 790 kb region. We thus put forward an approach that could rapidly fine map QTLs using only a large F_2_ population, especially for those traits governed by single nuclear-encoded genes or some threshold traits (Fig. 5). Developing a large F_2_ population, mapping by sequencing analysis and KASP genotyping for QTL mapping could be completed in a short time, and a progeny test could ensure accurate phenotype identification and helps in achieving the desired objectives rapidly and accurately. We believe that this approach can be adopted for quick QTL fine mapping in the future.

### The characteristics of the potential candidate gene *Solyc01g007130.3*

We used ProtComp 9.0 for the sub-cellular localization of *Solyc01g007130.3* and found that the encoded product was located in the plasma membrane (Additional file 3: Table S6), in agreement with other Cf genes. The CDD online protocol (https://www.ncbi.nlm.nih.gov/Structure/cdd/wrpsb.cgi) predicted that *Solyc01g007130.3* encodes a member of the STKc_IRAK-like superfamily and the PKc-like superfamily with 10 LRRs associated with disease resistance [61]. To further investigate the relationship between *Solyc01g007130.3* and other Cf genes, we used MEGA 5.05 to perform multiple DNA sequence alignment analyses. A long evolutionary distance was observed between *Solyc01g007130.3* and other Cf genes (*Hcr2*, Cf-2, Cf-4, Cf-5, Cf-9) (Additional file 4: Figure S2, A), strongly suggesting that comparatively different resistance mechanisms underlie Cf*-10* gene. Amino acid sequence clustering also support these results. More follow-up experimental investigation is required to further understand the molecular function of *Solyc01g007130.3.*

### The casual variation in Cf*-10* responsible for disease resistance

Most plant disease resistance genes (*R* genes) cloned so far encode NBS-LRR proteins [27, 28]. Therefore, when we located the Cf*-10* gene in the 790-kb interval, we only screened for genes containing the LRR domain. Fortunately, we only found one gene containing this domain (*Solyc01g007130.3*), and RT-qPCR analysis showed that significant or highly significant expression differences existed at 9 dpi and 12 dpi. However, *Solyc01g007130.3* was not included in the set of DEGs at 16 dpi in our previous transcriptome research [44]. This phenomenon may be explained by the fact that *Solyc01g007130.3* expression was significantly higher in the early stage of infection and that, in the later stage, downstream regulation played a leading role, but the hypothesis requires validation.

We also compared the parental re-sequencing data and found that no SNP or InDel variation was observed between the two parents. To better understand this step, we used Sanger sequencing to compare *Solyc01g007130.3* between the parents and found that no difference in this gene existed between the parents (data not shown). We did not rule out the possibility of a methylation modification difference between the parents [62] and also compared the 5 kb region upstream of this gene. Unfortunately, this region could not be cloned by traditional PCR. Using reference genome information available for tomato (ftp://ftp.solgenomics.net/tomato_genome/assembly/build_3.00/), we found that the GC content of this 5 kb region upstream of *Solyc01g007130.3* was 24.14%, which was much lower than the average value (35.20%) of the entire genome. This could be the possible reason hindering PCR based cloning of the region. In future research, we plan to establish the bacterial artificial chromosome (BAC) library for Ontario 792 to solve this problem of cloning the 5 kb sequence upstream of *Solyc01g007130.3*. Recently, a new method for cloning R genes that utilizes a three-step method (MutRenSeq) combining chemical mutagenesis with exome capture and sequencing to clone genes with specific domains may solve the problems associated with cloning the Cf*-10* gene [63].

## Conclusion

We used two steps of bioinformatic analysis named SNP-index and InDel-index to co-location and narrow down the Cf*-10* gene region to 3.29 Mb. Then QTL mapping analysis adopting a total of five KASP markers developed by SNPs in this region further mapped Cf*-10* gene to 790 Kb. This combinatorial approach can rapidly map or fine map genes responsible for specific traits just utilizing a big F_2_ population. Furthermore, we screened possible candidate genes according to their annotation information and found that *Solyc01g007130.3* harbored a receptor protein kinase TMK1 with a LRR motif. Different expression by RT-qPCR validated that *Solyc01g007130.3* was a candidate gene for Cf*-10*. The location and candidate gene screening of Cf*-10* could lay a robust foundation for later cloning the Cf*-10* gene and applications in MAS selection programs.

## Methods

### Plant materials and breeding strategy

In order to map the Cf*-10* gene in tomato using map-based strategy, we crossed accession Ontario 792 (Institute of Vegetable and Flowers, Chinese Academy of Agricultural Science) harbouring the Cf*-10* resistant gene (R gene) with the susceptible accession Moneymaker (Tomato Genetic Resource Center, LA2706) to harvest F_1_ and an F_2_ population subsequently containing 529 individuals, generated by selfing F_1_ population. At the same time, a BC_1_F_1_ population was also generated by back-crossing the F_1_ plants with Moneymaker to test the genetic basis of the Cf*-10* gene. All plants were grown at the Horticultural Experimental Station of Northeast Agricultural University by applying normal water and fertilizer practices while weeds were managed manually.

### *Cladosporium fulvum* inoculation and resistance evaluation

Ten physiological races (1.2, 1.2.3, 1.2.3.4, 1.2.4, 1.3.4, 2.3, 1.3, 1.4, 1.2.3.4.5, 1.2.3.4.9) provided by tomato research group of Horticulture College, Northeast Agricultural University were used to test the resistance potential of Ontario792 and Moneymaker. The disease score (0–9 points) for the severity of symptoms was assessed according to Zhao et al.’s method [38]. The disease index and the classification criteria were judged as per Li et al. [64] recommendations.

At five-to-six leaf stage, the seedling of Ontario 792 (50 pure lines), Moneymaker (50 pure lines), F_1_, F_2_ and BC_1_F_1_ plants were inoculated with *C. fulvum* race 1.2.3.4, which is a predominant physiological race in Heilongjiang Province, China. To ensure that the leaves of each individual were successfully inoculated, we sprayed the *C. fulvum* suspension at 1 × 10^7^ sporangia per mL in the morning, afternoon and evening of a sunny day as mentioned by Liu et al. [44]. The plants were assessed for disease severity as described by Wang et al. [65]. The disease severity of the plants was converted to a disease score of 0–9 points according to the method of Zhao et al. [38]. Scoring in the range of 0–3 points was demarcated as resistant and a score ≥ 5 points was defined as susceptible. For the subsequent linkage analysis, the resistant and susceptible phenotypes were assigned as 1 and 0, respectively. *Chi-square* analysis was performed to test the segregation ratio of the resistant and susceptible individuals of F_2_ population and BC_1_F_1_ population.

### DNA extraction, library construction and whole-genome re-sequencing

Using a minor modified cetyltrimethylammonium bromide (CTAB) method [66], all young leaves of 529 F_2_ individuals were harvested separately for total genomic DNA extraction. Of these, 20 resistant and 20 susceptible plants from the F_2_ population and the two parents were chosen for library construction and whole-genome re-sequencing. To simplify the description in later parts of this paper, we abbreviate Ontario 792 as R01, Moneymaker as R02, resistant pool as R03 and susceptible pool as R04. The isolated DNA was quantified using a NanoDrop 2000 spectrophotometer (Thermo Scientific, Fremont, CA, USA). All 20 resistant and 20 susceptible plants were precisely quantified on Qubit® 2.0 Fluorometer (Life Technologies, CA, USA). Equal amounts of DNA from the resistant and susceptible plants were mixed to prepare R03 and R04. These two samples along with R01 and R02 were sonicated to generate 350-bp fragments using S2/E210 Ultrasonicator (Covaris, Woburn, MA, USA); which were subsequently end repaired and nucleotide (A) overhangs were generated. Afterwards, sequencing adapters were ligated using T4 DNA ligase and PCR was performed. The PCR products were then purified and loaded onto an Illumina sequencing platform (Illumina, Inc., San Diego, CA, USA) for paired-end sequencing according to the manufacturer’s recommendations.

### QTL-seq and linkage mapping analysis

The raw sequencing data were filtered [reads with more than 50% of bases having Q-value ≤10 or an ambiguous sequence content (“N”) exceeding 10%] using an in-house Perl script available with Biomarker Technologies Co. Ltd. (Beijing, China). Then, these high-quality data were mapped onto the *Solanum lycopersicum* genome sequence (ftp://ftp.solgenomics.net/tomato_genome/assembly/build_3.00/) using a Burrows-Wheeler Aligner (Li et al. 2009). Picard software (https://sourceforge.net/projects/picard/) was used to mark duplicates. The SNP and InDel (1–5 bp) calling were realized by GATK [67] using default settings. In order to obtain highly accurate SNP and InDel set, a range of filters were also employed [68]. Both SNP-index [69] and InDel-index [17] methods were used for the association analysis. The SNP-index dot loess was obtained by regression fitting as described by Abe et al. [69]. The threshold value to map Cf*-10* resistant gene controlled by a dominant locus was expected to be 0.667 for an F_2_ population.

Using information from the QTL-seq analysis on the SNPs in the QTL region, a total of 16 KASP markers were designed using Primer 5.0 (Additional file 5: Table S1) to genotype 147 individuals, including 141 from F_2_ and two from each of F_1_, Ontario 792 and Moneymaker individuals respectively. The KASP assays were performed in a 1536-well plate format following the protocol of LGC Genomics (LGC, Middlesex, UK). The KASP reaction mixture system is shown in Additional file 6: Table S2. A S1000TM Thermal Cycler PCR (Bio-Rad, Hercules, CA) was used with the following settings: thermal activation at 95 °C for 15 min, denaturation at 95 °C for 20 s, primer annealing at 65 °C for 60 s (decreasing by 1 °C per cycle, 10 cycles totally) and finally 30 cycles of amplification (95 °C for 10 s; 57 °C for 60 s). The Synergy H1 full-function microplate reader (FLUOstar Omega, BMG Labtech, Germany) was used to read the fluorescence signal upon completion of the reaction.

### Real time quantitative PCR (RT-qPCR) analysis

The RT-qPCR assay was carried out in order to explore the expression pattern of candidate genes in Ontario 792 and Moneymaker at 0, 5, 9, 12 and 16 days post-infection (dpi). The leaves from Ontario 792 and Moneymaker individuals were sampled independently for total RNA extraction using Trizol (Invitrogen) as per manufacturer’s recommendations. The primers for candidate genes were designed using primer 5.0 software. Actin-EFα1 was used for internal normalization. The primer sequences and reaction system are shown in Additional files 7 and 8: Tables S3 and S4, respectively and the RT-qPCR was performed in an iQ5 system (Bio-Rad, USA). A 20 μL reaction mixture, including 2 μL of cDNA (1:10 dilution), 10 μL of 2× TransStart Top Green qPCR SuperMix (TransGen, China), 0.5 μL of each primer (10 μmol/μL) and ddH_2_O, was prepared. Reaction conditions were as follows: 95 °C for 7 min; 40 cycles at 95 °C for 10 s, 58 °C for 30 s, and 72 °C for 20 s and then 71 cycles at 95 °C for 10 s, 60.5 °C for 10 s, and 95 °C for 10 s. The relative gene expression levels were calculated using the 2^−ΔΔCt^ method [70].

## Additional files

Additional file 1: **Table S5.** The KASP genotyping results. (XLSX 16 kb)

Additional file 2: **Figure S1.** QTL mapping analysis by Icimapping software. (PDF 36 kb)

Additional file 3: **Table S6.** The sub-cellular location prediction of Cf*-10*. (XLSX 14 kb)

Additional file 4: **Figure S2.** Cluster analysis of candidate and other genes. A Cluster analysis on account of DNA sequence. B Cluster analysis on account of amino acid sequence. (PDF 51 kb)

Additional file 5: **Table S1.** The primer information of KASP markers. (XLSX 11 kb)

Additional file 6: **Table S2.** The KASP reaction system. (XLSX 10 kb)

Additional file 7: **Table S3.** The primer information of EFα1 and candidate gene. (XLSX 10 kb)

Additional file 8: **Table S4.** The qRT-PCR reaction system. (XLSX 10 kb)

## Abbreviations

BSA: bulked segregant analysis
CAPS: cleaved amplified polymorphic sequence
DEGs: differentially expressed genes
DH: double-haploid
InDel: insertion-deletion
KASP: kompetitive allele-specific PCR
MAS: marker-assisted selection
NBs-LRR: nucleotide-binding sites and leucine-rich repeat
NGS: next-generation sequencing
NIL: near-isogenic line
QTL: quantitative trait locus
RIL: recombinant inbred line
RT-qPCR: real time quantitative polymerase chain reaction
SCAR: sequence-characterized amplified region
SNPs: single nucleotide polymorphisms
SSR: simple sequence repeat
