# Supplementary figures and images for: Molecular mapping of the Cf-10 gene by combining SNP/InDel-index and linkage analysis in tomato (Solanum lycopersicum)
Source: BMC Plant Biol. 2019 Jan 8;19:15. doi: 10.1186/s12870-018-1616-7 (PMC6325758; doi:10.1186/s12870-018-1616-7)

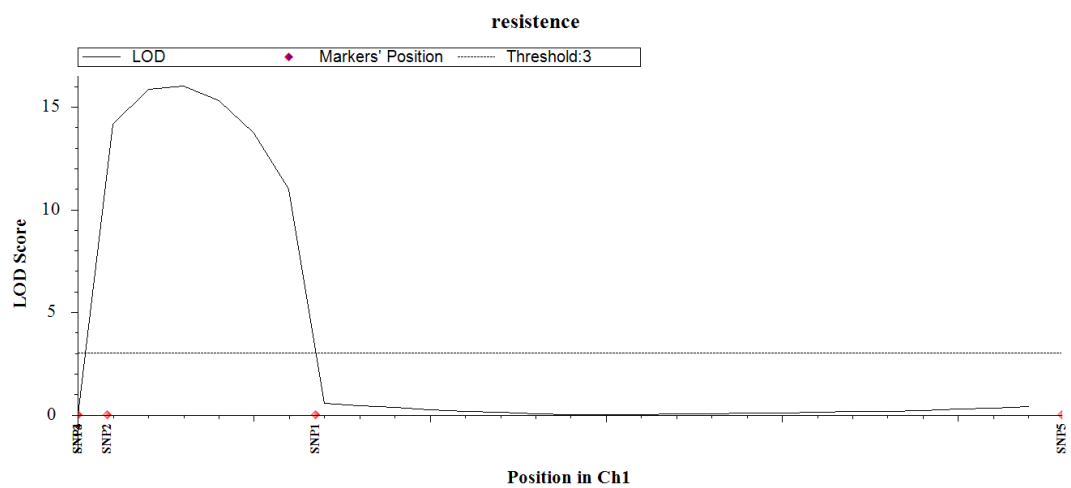

Supplement: Supplementary file 2 — Figure S1. QTL mapping analysis by Icimapping software. (PDF 36 kb) [file 12870_2018_1616_MOESM2_ESM.pdf]

A

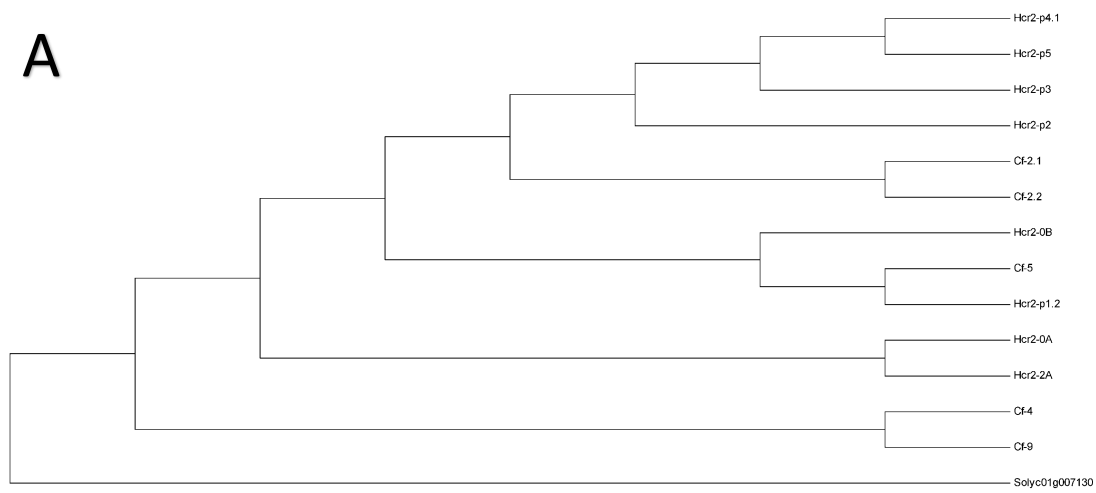

B

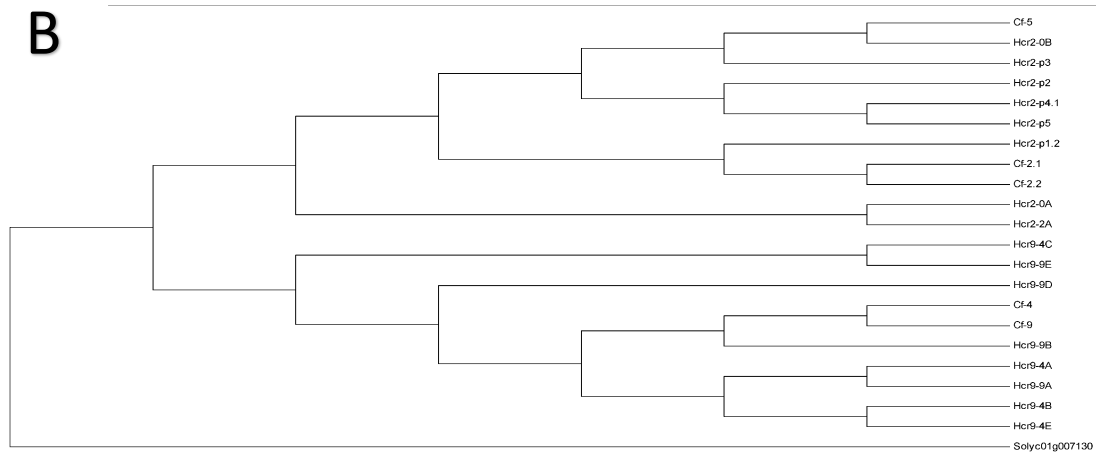

Supplement: Supplementary file 4 — Figure S2. Cluster analysis of candidate and other genes. A Cluster analysis on account of DNA sequence. B Cluster analysis on account of amino acid sequence. (PDF 51 kb) [file 12870_2018_1616_MOESM4_ESM.pdf]
